# *In-vivo* absorption of pinocembrin-7-*O-β*-D-glucoside in rats and its *in-vitro* biotransformation

**DOI:** 10.1038/srep29340

**Published:** 2016-07-05

**Authors:** Wei-Wei Guo, Feng Qiu, Xiao-Qing Chen, Yin-Ying Ba, Xing Wang, Xia Wu

**Affiliations:** 1 Beijing Key Lab of TCM Collateral Disease Theory Research, School of Traditional Chinese Medicine, Capital Medical University, 10 Youanmen, Xitoutiao, Beijing 100069, China

## Abstract

Pinocembrin-7-*O-β*-D-glucoside (PCBG), a flavonoid isolated from *Penthorum chinense* Pursh., has significant liver-protecting effects. The pharmacokinetics of PCBG and its major metabolite pinocembrin (PCB) in rats were investigated in this study. A sensitive and accurate UPLC-MS/MS method was developed and validated for the simultaneous quantitative determination of PCBG and PCB in rat plasma after oral and intravenous administration of PCBG. After intravenous administration, PCBG was the main form in plasma. In contrast, after oral administration, the concentration of PCB was about 4-fold higher than that of PCBG, indicating that PCBG was metabolized to PCB. We also investigated the biotransformation of PCBG *in vitro* in order to understand whether the pH and the intestinal flora of gastrointestinal tract could affect the metabolism of PCBG. PCBG was incubated in rat plasma, liver homogenization, gastrointestial contents, liver microsomes (RLM) and hepatocytes *in vitro*. The data showed that PCB was quickly formed in the gastrointestinal incubation but PCBG was converted to PCB gradually in other incubations. The results indicated that the majority of PCBG was converted to its aglycone PCB in digestive system after oral administration, and PCB could be the active ingredient in the body.

*Penthorum chinense* Pursh. (*P. chinense*) belongs to the family of Saxifragaceae, which is recorded in the Dictionary of Chinese Materia Medica as a species of Miao nationality^1^. Previous studies have shown that extracts from *P. chinense* have antioxidant and anti-complement effects^2^. Therefore, it is commonly used as the traditional medicine as well as food supplement for protecting liver^3^. The hepatoprotective effect of extracts from *P. chinense* is mainly related to its activities in modulation of liver enzymes^4^ and free-radical scavenging^5^. In our previous research, pinocembrin-7-*O-β*-D-glucoside (PCBG) was isolated as one of the major flavonoids in *P. chinense*^6^,^7^. The concentration of PCBG in the plant extracts was quantified by high performance liquid chromatography with diode array detection (HPLC-DAD), which was found to be higher than its aglycone, pinocembrin (PCB)^8^. PCBG and PCB are reported to have hepatoprotective, antimicrobial, anti-inflammatory, antioxidant and antihepatocarcinoma activities as well as neuroprotective effects^9^,^10^.

Based on previous reports^11^, the aglycone PCB was suggested to be the metabolic product of flavonoid glycoside PCBG by biotransformation *in vivo*. Since the pharmacological activities of metabolites are related to their safety and efficacy, great attention has been paid on the metabolism of active ingredients in Traditional Chinese Medicine (TCM)^12^. However, the pharmacokinetics property of PCBG has not been reported.

In this study, a sensitive and accurate UPLC-ESI-MS/MS method was developed and validated to quantitatively detect PCBG and PCB simultaneously in rat plasma, and the method was applied to a pharmacokinetic (PK) study in rats after oral and intravenous administration of PCBG. As expected, PCB was detected in rat plasma. Especially after oral administration, the concentration of PCB was about 4-fold higher than that of PCBG. Moreover, the metabolic stability of PCBG in various rat fluids and tissues was tested *in vitro*. The results showed that the majority of PCBG was degraded into PCB in a short time after incubation in the gastrointestinal content, which could explain its oral pharmacokinetic behavior.

## Results

### UPLC-MS/MS method development

**Figure 1:**
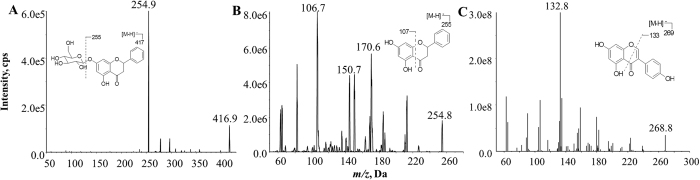
Full scan product ion spectra of [M-H]^−^ ions of (**A**) PCBG, (**B**) PCB and (**C**) genistein(IS).

Both PCB and PCBG produced strong signals in the negative ion mode due to the presence of a phenolic hydroxyl group in their structures. Genistein was selected as internal standard (IS), because it showed strong MS response under negative ion mode and presented chromatographic behavior similar to those of PCBG and PCB^13^. Full-scan product ion spectra of the [M-H]^−^ ions and fragmentation pathways for PCB, PCBG and IS are shown in Fig. 1. Mass spectrometry detection was performed in negative ESI mode and the data were acquired in multiple reaction monitoring (MRM) mode.

### Method validation

The UPLC-ESI-MS/MS method was validated for selectivity, linearity, lower limit of quantification (LLOQ), intra-day precision, inter-day precision, accuracy, extraction recovery and stability according to US Food and Drug Administration (FDA) guidelines for bioanalytical method validation^14^.

**Figure 2:**
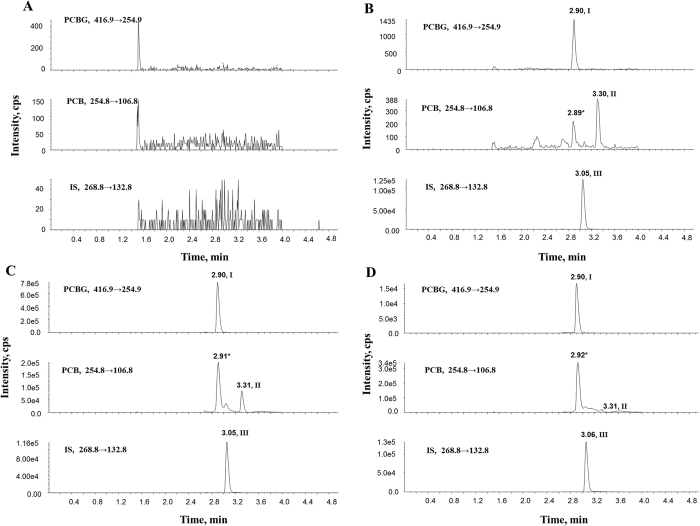
Typical MRM chromatograms of (**A**) blank rat plasma; (**B**) blank plasma spiked with PCBG (5 ng/mL), PCB (5 ng/mL) and IS; (**C**) rat plasma sample at 10 min after oral administration of PCBG at 50 mg/kg spiked with IS; and (**D**) rat plasma sample at 10 min after intravenous administration of PCBG at 10 mg/kg spiked with IS. Peak I, PCBG; Peak II, PCB; Peak III, genistein.

The selectivity was tested by comparing the chromatograms of six batches of blank rat plasma with corresponding spiked plasma samples. The typical MRM chromatograms of a blank rat plasma sample, a blank rat plasma spiked with PCBG (5 ng/mL), PCB (5 ng/mL) and IS, a rat plasma sample after oral administration of 50 mg/kg PCBG, and a rat plasma sample after intravenous administration of 10 mg/kg PCBG are shown in Fig. 2. There were no significant interferences at retention times of 3.05 (IS), 3.32 (PCB) and 2.91 min (PCBG). The detection of PCB, PCBG and genistein by MRM was highly selective with no significant interferences. The run time was set at 5 min because full chromatographic separation was also necessary to avoid potential matrix effect.

The calibration curves were obtained and fitted over the concentration range of 1–5000 ng/mL assayed in duplicate on three separate days, which were linear to analyze both PCBG and PCB in rat plasma. Plasma samples were quantified using the ratio of the peak area of each analyte to that of genistein. The calibration equation of the peak area ratio (*y*) versus concentration of each analyte (*x*) and correlation coefficients were calculated using weighted (1/*x*^2^) least squares linear regression analysis. The regression equations determined by least squares linear regression using a nine-factor of 1/*x*^2^ were *y* = 2.03 × 10^−3^ *x* + 5.13 × 10^−4^ (r = 0.9978) for PCBG and *y* = 6.80 × 10^−4^ *x* + 6.47 × 10^−5^ (r = 0.9968) for PCB. The regression coefficients (r) were higher than 0.995, showing a good linearity among the concentration range. The lower limit of quantification (LLOQ) was 1.0 ng/mL for both PCBG and PCB, which was sufficiently sensitive for the determination of the low concentrations of the analytes in rat plasma.

Quality control (QC) samples were prepared with blank plasma at three concentration levels of 2.0, 100 and 4000 ng/mL. QC samples at three concentrations in six replicates were analyzed on three different days to determine the intra- and inter-day precision (% relative standard deviation, RSD) and accuracy (% relative error, RE). The results of precisions and accuracies are presented in Table 1. The precisions of the low-level quality control (QC) samples were less than 8.1% (RSD) and the precisions of the high and medium levels were less than 7.0% (RSD), and the relative errors were from −0.8% to 3.5%. In summary, all the values were within the accepted limits and the precision and accuracy results showed reliability for determining the contents of PCBG and PCB.

As shown in Table 2, the recoveries of the investigated components in plasma at three different concentration levels were 78.0–87.2% for PCBG and 89.2–93.3% for PCB. The results of matrix effect were 89.7–94.6% for PCBG and 86.7–89.4% for PCB. The results suggested that the matrix effects for analytes were in acceptable range.

The stability of PCB and PCBG in rat plasma at three QC concentration levels was investigated under different storage and processing conditions. The long-term stability at −20 °C was evaluated for 30 days. The short-term room temperature stability was investigated at room temperature for 24 h. The freeze-thaw stability was assessed by three consecutive freeze (−20 °C) − thaw (20 °C) cycles. The results are shown in Table 3. PCB and PCBG in rat plasma were found stable after stored at 20 °C for 24 hours, stored at −20 °C for 30 days and three freeze-thaw cycles. It was indicated that the method provided good stability with the relative error (% RE) in the range of −13.5% to 6.1%.

### Pharmacokinetic study

**Figure 3:**
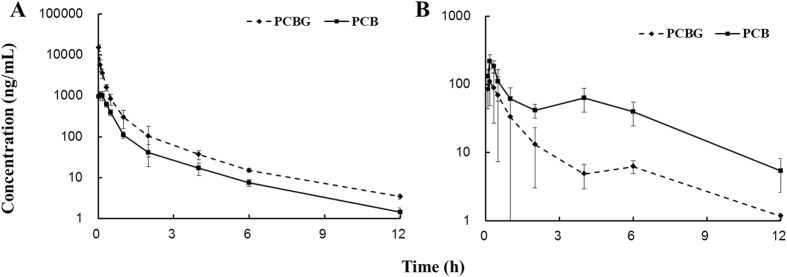
Plasma concentration-time profile of PCBG and PCB after (**A**) intravenous administration of 10 mg/kg PCBG and (**B**) oral administration of 50 mg/kg PCBG to rats. Values of each point was expressed as mean ± SD (n = 5).

The developed method was successfully applied in a PK study of PCBG in Sprague-Dawley rats after oral and intravenous administration of PCBG. The mean plasma concentration-time profiles of PCBG and its metabolite PCB are shown in Fig. 3. The main PK parameters for both oral and intravenous administration are listed in Table 4. The results showed that PCB could be detected in plasma after oral and intravenous administration of PCBG, which indicated that PCBG would convert to its aglycone PCB *in vivo*. After an oral administration of 50 mg/kg PCBG, the mean peak plasma concentration (*C*_max_) of PCBG and PCB were 109.0 and 234.4 ng/mL, respectively. The times reaching peak plasma concentration (*T*_max_) of PCBG and PCB ranged from 0.15 h to 0.23 h following oral administration, which suggested moderately rapid absorption pattern, while half-time (*t*_1/2_) of PCBG and PCB were 2.5 ± 0.0 h and 3.1 ± 1.2 h, respectively. And the value of the area under concentration-time curve (AUC_0−t_) was 137.6 (h ng/mL) for PCBG as well as 517.9 (h ng/mL) for PCB. For intravenous administration, the plasma concentration of PCBG decreased sharply during the first 30 min after injection, which could suggest that PCBG might be transformed into PCB in the liver quickly. The *t*_1/2_ of PCBG and PCB were 5.0 ± 2.2 h and 2.1 ± 0.7 h, respectively. Their AUC_0−t_ were 2815.8 and 686.1 (h ng/mL), respectively. The absolute bioavailability (F %) of PCBG for oral was estimated to be 0.95%, based on the calculation of AUC_0−t_ obtained from oral and intravenous administration.

### *In vitro* biotransformation of PCBG

**Figure 4:**
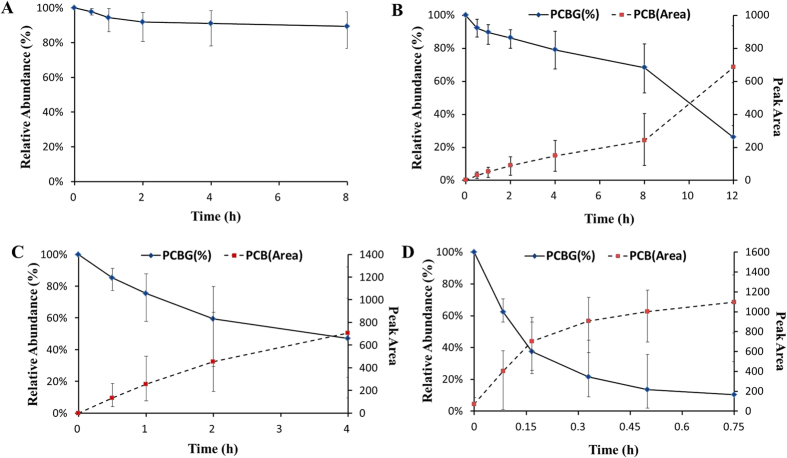
Relative abundance of PCBG in (**A**) plasma,(**B**) liver homogenization, (**C**) gastric contents and (**D**) small intestinal contents incubations.Values of each point was expressed as mean ± SD (n = 3).

The percentage of remaining PCBG was determined by HPLC-DAD analysis using the substrate peak area at *t* = 0 min as 100%, which was plotted versus incubation time. Only the initial time points were used to fit a first-order decay function, wherein log-linearity was observed, and the *t*_1/2_ was calculated using the following equation: *t*_1/2_ = 0.693/*k* (*k* is the substrate depletion rate constant). Figure 4 showed the metabolism of PCBG, which indicated PCBG was metabolized to PCB as evidenced by the relative abundance-time profiles. The degradation rate of PCBG incubated in plasma for 8 h was 10.7% and *t*_1/2_ was 130.8 h (Fig. 4A). There was no PCB detected by UPLC-MS/MS. However, PCBG was degraded obviously in liver homogenization (Fig. 4B). At 12 h, only 26% PCBG remained and the *t*_1/2_ was 16.7 h. When incubated with RLM and hepatocytes, less than 5% PCBG was degraded at 4 h and the *t*_1/2_ was about 216.6 h. As PCBG showed very low oral bioavailability and PCB appeared quickly in PK study after oral administration, especially at a higher concentration than PCBG, it was necessary to investigate the biotransformation of PCBG in gastric content. Figure 4C showed that PCBG was degraded quickly in gastric content. More than half of the substrate was consumed at 4 h, and the *t*_1/2_ was 6.2 h. The concentration of PCB increased with the decrease of PCBG concentration. Most of PCBG was converted to PCB within one hour when incubated with intestinal content (Fig. 4D), and the *t*_1/2_ in small intestinal content was only 0.53 h.

## Discussion

In this study, a sensitive, accurate and rapid UPLC-ESI-MS/MS method was developed and validated for the simultaneous determination of PCBG and PCB in rat plasma, and it was applied in a pharmacokinetic study of PCBG by oral and intravenous administration for the first time. The results demonstrated that the oral bioavailability (F %) of PCBG was estimated to be 0.95%. Following oral administration of PCBG, double peaks were observed in the curves of mean plasma concentration for PCBG and PCB. This phenomenon has been often observed in the pharmacokinetics study of Chinese herbal medicines^15^,^16^. It may be attributed to the distribution, re-absorption and exterohepatic circulation^17^,^18^. The concentration of PCB was about 4-fold higher than that of PCBG after oral administration, which indicated that PCBG was rapidly transformed into its metabolite PCB. It is well known that flavonoid glycosides can be converted to their aglycones in the intestinal tract by the hydrolytic action of bacteria^19^,^20^,^21^,^22^. And aglycones are the main metabolites absorbed into the body^23^. For example, it was reported that esculin and its metabolite esculetin were detected simultaneously after oral administration of esculin, and esculin and its hydrolysate were detected instantly from rat plasma^12^.

Liver microsomes and primary hepatocytes are useful tools in drug metabolism assessments^24^,^25^ and commonly used for biotransformation studies^26^,^27^,^28^. The remaining PCBG was 96.8% and 97.5% after 2 h incubation with RLM and hepatocytes, respectively, and the incubation did not generate any detectable metabolites. In liver homogenate, PCBG was metabolized slowly and only degraded by 20% after 4 h incubation. Therefore, PCBG was relatively stable in liver homogenate and liver microsomes.

The research has studied that both phase I and phase II metabolic enzymes were expressed in the intestinal mucosa^29^. Uptake as well as metabolism of orally administered drugs occurs in the small intestine. Metabolism in intestine leads to low bioavailability of oral drugs. Our study showed the *t*_1/2_ of PCBG in plasma was 130.8 h, indicating PCBG was relatively stable in plasma. However, when incubated with gastrointestinal contents, PCBG was converted to its aglycone PCB rapidly. Especially when incubated with intestinal content, PCBG was metabolized to PCB completely within 1 h. Therefore, the majority of PCBG was degraded into PCB in gastrointestinal tissues once administered. This phenomenon can be explained by the rapid degradation of flavonoid glycosides to their aglycones in the gastrointestinal tract by the acidic environment or by the hydrolytic actions of bacteria and enzymes^21^,^22^. The metabolite PCB was absorbed into blood and it could be the main active ingredient for the biological activities of *P. chinense*.

## Methods

### Chemicals and reagents

Reference standards of PCB and PCBG (purity both ≥98% by HPLC at 280 nm, identified by ^1^H-NMR, ^13^C-NMR) were prepared in the Laboratory of Chemistry of Chinese Medicinal Materials of Capital Medical University (Beijing, China). Genistein (Internal standard, IS) was provided by Shenyang Pharmaceutical University (Shenyang, China). HPLC grade acetonitrile and methanol were obtained from TEDIA Company (Fairfield, OH, USA). Cryopreserved hepatocytes from Spague-Dawley (SD) rats and rat liver microsomes were purchased from XenoTech LLC (Lenexa, KS, USA). *β*-Nicotinamide adenine dinucleotide 2′-phosphate reduced tetrasodium salt (NADPH) and dimethyl sulfoxide (DMSO, BioReagent, ≥99.7%) were purchased from Sigma-Aldrich Co. (St. Louis, MO, USA). Dulbecco’s Modified Eagle Medium (DMEM) and fetal bovine serum (FBS) were purchased from Invitrogen (Grand Island, NY, USA). All other chemicals were of analytical grade. Distilled water was prepared by a Milli-Q water purification system from Millipore (Molsheim, France).

### Animals and drug administration

Male Sprague-Dawley (SD) rats (190–220 g) were provided by the Vital River Laboratories (VRL) (Beijing, China). The experimental procedures were carried out following the guidelines of the Experimental Animal Care and Use Committee at the Capital Medical University (Beijing, China). All studies were approved by the Capital Medical University Animal Experiments and Experimental Animals Management Committee (IACUC Protocol No 2012-X-79). For *in vivo* assay, a total of 10 rats (n = 5 in each group) were fasted for 12 h prior to administration, but with free access to water. Two days before the experiment, polyethylene cannula was implanted in the femoral vein of rats anesthetized with pentobarbital (50 mg/kg, intravenous). The cannula were externalized at the back of the neck and filled with heparinized saline (20 units/mL).

PCBG was freshly prepared at the concentration of 5 mg/mL and homogenously suspended in 0.5% sodium carboxyl methyl cellulose (CMC-Na) for oral administration, and dissolved in DMSO:PEG400:PG:water (5:20:25:50, v/v/v/v) at a final concentration of 2 mg/mL for intravenous administration. For *in vitro* assays, PCBG was dissolved in DMSO at a concentration of 200 mM as a stock solution.

### UPLC-ESI-MS/MS

The UPLC-ESI-MS/MS system was used in the measurement of concentrations of PCBG and its bio-product *in vivo*. Analyses were acquired on a Shimadzu LC-20AD series UPLC system (Shimadzu, Japan) coupled with an Applied Biosystems Sciex Q-trap 4000 mass spectrometer (Concord, Ontario, Canada) with electrospray ionization (ESI) and atmospheric pressure chemical ionization (APCI) sources. The LC-MS/MS system was controlled and data were processed with Analyst software version 1.5.2. PCBG, PCB and IS were separated on an Agilent Zorbax XDB C18 column (50 mm × 2.1 mm, 3.5 μm, USA). The mobile phase consisted of 0.1% formic acid aqueous solution (A) and 0.1% formic acid acetonitrile solution (B). The gradient elution was as follows: 0–0.6 min, 2% B; 0.6–2.5 min, 2% B–98% B; 2.5–3.5 min, 98% B; 3.5–3.51 min, 98% B–2% B; 3.51–5 min, 2% B. The column temperature was maintained at room temperature (22 ± 2 °C) with a flow rate of 0.45 mL/min. Under these chromatographic conditions, the retention times of PCB and PCBG were 3.32 and 2.91 min, respectively. The total chromatographic running time per sample was 5 min.

Mass spectrometry detection was performed in negative ESI mode and the data were acquired in multiple reaction monitoring (MRM) mode. Nitrogen was used as the nebulizer, heater, and curtain gas, as well as the collision activation dissociation (CAD) gas. The capillary temperature was set at 550 °C. The ionspray voltage was −4200 V. Nebulizer (GS1), heater (GS2), and curtain gas flow rates were set at 60, 50, and 20 units. The precursor-to-product ion transitions were monitored at *m/z* 416.9 → 254.9 with collision energy (CE) of −25 eV for PCBG, *m/z* 254.8 → 106.8 with CE of −38 eV for PCB and *m/z* 268.9 → 132.8 with CE of −40 eV for IS.

### HPLC conditions

HPLC analysis was performed on an Agilent series 1260 HPLC instrument (Agilent, Germany) consisting of a quaternary pump, a Diode Array Detector (DAD), an autosampler and a column compartment. Chromatographic separations were performed on an Agilent Zorbax SB C18 column (250 mm × 4.6 mm, 5 μm, USA). The mobile phase consisted of acetonitrile (B) and 0.2% (v/v) formic acid in water (A) with a gradient of 10–50% B at 0–65min, 50–10% B at 65–70 min. The analysis was performed at 30 °C with a flow-rate of 1 mL/min. The detection wavelength was 280 nm. An aliquot of 20 μL was injected for HPLC. The data were processed with Agilent Technologies ChemStation Revision B.04.03 software.

### Animal studies

Ten rats were randomly divided into two groups, oral and intravenous administration. After a 2-day recovery from femoral vein cannulated surgery, five rats were orally administered with 50 mg/kg dose of PCBG, and the other rats (five) were injected with 10 mg/kg dose of PCBG at a final concentration of 2 mg/mL. After administration, approximately 0.15 mL blood samples were collected into heparinized 1.5 mL polythene tubes at different time points of 0.083, 0.17, 0.33, 0.5, 1, 2, 4, 6 and 12 h for oral administration and 0.033, 0.083, 0.17, 0.33, 0.5, 1, 2, 4, 6 and 12 h for intravenous administration. Plasma was separated by centrifugation at 5000 × *g* for 10 min. 50 μL aliquots of plasma were mixed with 150 μL of IS solution (400 ng/mL) and 5 μL methanol (or a standard or QC solution) were added. The samples were centrifuged at 5000 × *g* for 10 min after vortexing for 1 min. A 10 μL aliquot of each supernatant was injected into the UPLC-ESI-MS/MS system for analysis.

### *In vitro* incubation

Incubation assays were carried out to investigate the biotransformation and metabolism of PCBG by incubating PCBG with fresh plasma, liver homogenization, RLM and hepatocytes, and gastrointestinal contents. Preparation of incubation systems were done in accordance with the research reported^30^. Gastric and small intestinal contents were collected by rinsing stomach and small intestine with HCl solution (pH = 1.2) and phosphate buffer solution (pH = 6.8) respectively. The protein concentration of gastointestinal content was adjusted to 1.2 g/L with corresponding solution. PCBG was dissolved with DMSO at the concentration of 200 mM as stock solution. PCBG solution was added into incubation systems at a final concentration of 40 μM in plasma, 200 μM in liver homogenization and gastrointestinal contents. After mixed, 0.2 mL incubated samples (n = 6) were removed at different time points of 0, 0.083, 0.17, 0.33, 0.5, 1, 2, 4 and 8 h, vortexed for 1 min after added 0.2 mL acetonitrile to end reaction and precipitate proteins. The samples were centrifuged at 5000 × *g* for 10 min. A 20 μL aliquot of each supernatant was injected into the HPLC system for analysis.

Rats liver microsomes were also used in the study. PCBG (50 μM) was incubated in triplicate in a reaction mixture containing 100 mmol/L potassium phosphate buffer (pH 7.4) and RLM at the final concentration of 0.5 mg/mL. After preincubation at 37 °C for 5 min, NADPH (1 mmol/L) containing 3.3 mmol/L MgCl_2_ was added to initiate the reaction. Each 200 μL was quenched with one volume of ice-cold acetonitrile at 0, 10, 20, 40, 80 and 120 min. The samples generated were directly analyzed by HPLC after centrifugation at 12000 × *g* for 10 min at 4 °C.

The metabolic stability of PCBG in cryo-preserved hepatocytes from rats (SD) was evaluated in triplicate. PCBG (10 μM) was incubated at a cell density of 1 × 10^6^ cells/mL in DMEM containing 10% FBS at 37 °C and under 5% CO_2_. Incubation was terminated by the addition of one volume of ice-cold acetonitrile at 0, 0.17, 0.5, 1, 2, 4 and 8 h. The samples generated were directly analyzed by HPLC after centrifugation at 5000 × *g* for 10 min at 4 °C.

### Statistical analysis

All experiments were conducted at least in triplicate sets. *In vivo* results were expressed as mean ± SD (standard deviation). Pharmacokinetic parameters including half-life (*t*_1/2_), maximum plasma time (*T*_max_) and concentration (*C*_max_), area under the concentration–time curve (AUC_last_ and AUC_Inf_), and mean residence time (MRT) of PCB and PCBG were analyzed with DAS Version 2.1.1 (Pharsight Corporation, Mountain View, USA).

## Additional Information

**How to cite this article**: Guo, W.-W. *et al*. *In-vivo* absorption of pinocembrin-7-*O-β*-D-glucoside in rats and its *in-vitro* biotransformation. *Sci. Rep*. **6**, 29340; doi: 10.1038/srep29340 (2016).

## Figures and Tables

**Table 1:** Intra- and inter-day accuracy and precision of PCBG and PCB in rat plasma (n = 3 days, 6 replicates per day).

Sample	Spiked (ng/mL)	Measured (mean ± SD)	Accuracy (RE%)	Precision (RSD%)
PCBG				
Intra-day	2	2.05 ± 0.13	2.6	6.5
	100	101.8 ± 7.10	1.8	7.0
	4000	3973 ± 235	−0.7	5.9
Inter-day	2	2.05 ± 0.12	2.4	6.0
	100	100.1 ± 5.73	0.1	5.7
	4000	3966 ± 233	−0.8	5.9
PCB				
Intra-day	2	2.06 ± 0.16	3.0	7.8
	100	102.7 ± 4.73	2.7	4.6
	4000	3979 ± 226	−0.5	5.7
Inter-day	2	2.01 ± 0.16	0.4	8.1
	100	103.5 ± 5.54	3.5	5.4
	4000	4061 ± 188	1.5	4.6

**Table 2:** The recoveries and matrix effects of PCBG and PCB in rat plasma (n = 6).

Spiked (ng/mL)	PCBG			PCB		
	Recovery(%)	RSD(%)	Matrix effect (%)	Recovery(%)	RSD(%)	Matrix effect (%)
2	87.2	1.3	93.2	89.2	3.3	86.7
100	84.0	2.2	94.6	91.1	0.6	89.4
4000	78.0	2.7	89.7	93.3	1.4	87.5

**Table 3:** Stability data of PCBG and PCB in rat plasma at different conditions (n = 3).

Conditions	Spiked (ng/mL)	PCBG			PCB		
		Measure (mean ± SD)	RSD (%)	RE (%)	Measure (mean ± SD)	RSD (%)	RE (%)
Room temperature (24 h)	2	1.88 ± 0.02	1.1	−6.2	1.95 ± 0.15	7.9	−2.3
	100	86.7 ± 1.26	1.5	−13.3	104.0 ± 2.48	2.4	4.0
	4000	3532 ± 30.4	0.9	−11.7	4227 ± 119	2.8	5.7
Storage at −20 °C (30 days)	2	1.91 ± 0.02	0.9	−4.6	1.95 ± 0.15	7.9	−2.3
	100	87.0 ± 2.79	3.2	−13.0	95.3 ± 0.81	0.9	−4.7
	4000	3462 ± 42.2	1.2	−13.5	3792 ± 157	4.1	−5.2
Three freeze-thaw cycles	2	1.87 ± 0.02	1.1	−6.7	2.12 ± 0.10	4.8	6.1
	100	90.6 ± 0.99	1.1	−9.4	97.6 ± 4.43	4.5	−2.4
	4000	3500 ± 41.4	1.2	−12.5	4035 ± 153	3.8	0.9

**Table 4:** Pharmacokinetic parameters of PCBG and PCB in rats after various treatments (n = 5, mean ± SD).

Parameters	Unit	Oral (50 mg/kg)		Intravenous (10 mg/kg)	
		PCBG	PCB	PCBG	PCB
AUC_(0-t)_	h ng/mL	137.6 ± 87.0	518 ± 170	2816 ± 180	686.1 ± 65.1
MRT_(0-t)_	h	2.55 ± 0.73	4.02 ± 0.57	0.72 ± 0.13	1.01 ± 0.18
*t*_1/2_	h	2.53 ± 0.32	3.11 ± 1.21	5.05 ± 2.17	2.14 ± 0.68
*T*_max_	h	0.15 ± 0.04	0.23 ± 0.09	—	0.10 ± 0.04
CL_z/F_	L/kg h	487.0 ± 206.2	110 ± 31.4	3.8 ± 0.3	15.5 ± 1.4
V_z/F_	L/kg	1840.3 ± 910.2	478 ± 213	26.8 ± 10.3	48.7 ± 19.6
*C*_max_	ng/mL	109.0 ± 59.9	234 ± 38.6	14920 ± 2923	1099.6 ± 257.8
*F*	—	0.95% ± 0.52%	—	—	—
